# Colorado and Its Climate

**Published:** 1881-04

**Authors:** 


COLORADO AND ITS CLIMATE is interesting many in the eastern Atlantic States, and the reports of the charmingly salubrious weather there during the past winter, are well calculated to attract the attention of persons suffering from lung troubles everywhere. The point which has received the largest influx of invalids thus far, is Colorado Springs; and from the fine accommodations the town affords, together with the climate, and valuable mineral water in its midst, it must continue to be a favorite resort for those in quest of health. Dr. B. P. Anderson has been identified with the *Springs* from its early settlement and would be glad to furnish any information that might be desired concerning that point as a health resort.

